# Structural Basis for the dsRNA Specificity of the Lassa Virus NP Exonuclease

**DOI:** 10.1371/journal.pone.0044211

**Published:** 2012-08-28

**Authors:** Kathryn M. Hastie, Liam B. King, Michelle A. Zandonatti, Erica Ollmann Saphire

**Affiliations:** 1 Department of Immunology and Microbial Science, The Scripps Research Institute, La Jolla, California, United States of America; 2 The Skaggs Institute for Chemical Biology, The Scripps Research Institute, La Jolla, California, United States of America; Centro de Biología Molecular Severo Ochoa (CSIC-UAM), Spain

## Abstract

Lassa virus causes hemorrhagic fever characterized by immunosuppression. The nucleoprotein of Lassa virus, termed NP, binds the viral genome. It also has an additional enzymatic activity as an exonuclease that specifically digests double-stranded RNA (dsRNA). dsRNA is a strong signal to the innate immune system of viral infection. Digestion of dsRNA by the NP exonuclease activity appears to cause suppression of innate immune signaling in the infected cell. Although the fold of the NP enzyme is conserved and the active site completely conserved with other exonucleases in its DEDDh family, NP is atypical among exonucleases in its preference for dsRNA and its strict specificity for one substrate. Here, we present the crystal structure of Lassa virus NP in complex with dsRNA. We find that unlike the exonuclease in Klenow fragment, the double-stranded nucleic acid in complex with Lassa NP remains base-paired instead of splitting, and that binding of the paired complementary strand is achieved by “relocation” of a basic loop motif from its typical exonuclease position. Further, we find that just one single glycine that contacts the substrate strand and one single tyrosine that stacks with a base of the complementary, non-substrate strand are responsible for the unique substrate specificity. This work thus provides templates for development of antiviral drugs that would be specific for viral, rather than host exonucleases of similar fold and active site, and illustrates how a very few amino acid changes confer alternate specificity and biological phenotype to an enzyme.

## Introduction

The causative agent of Lassa Fever, an arenavirus called Lassa, is endemic in Western Africa, causes hundreds of thousands of infections per year, and is the viral hemorrhagic fever most frequently transported to the United States and Europe. The nucleoprotein NP of Lassa virus, and other members of the arenavirus family, suppresses host innate immune signaling pathways [1], and may be linked to the profound immune disregulation noted in Lassa Fever infection [2].

Recent structural analysis identified a previously unknown exonuclease function for NP, placing it within the DEDDh exonuclease superfamily [3], [4]. Lassa NP has a tertiary fold that is similar to, and an exonuclease active site that is completely conserved with other DEDDh exonucleases like interferon-stimulated gene-20 (ISG-20) [5], *E. coli* DNA polymerase IIIε (ε186) [6], and Trex 1 [7], [8] and Trex 2 [9]. Yet among this family of enzymes, Lassa NP is the only one known to have a strict specificity for a certain substrate, and the only one known to efficiently digest double-stranded RNA [3]. For example ISG-20 can digest both ssRNA and DNA [10] while ε186 and Trex 1 and 2 can digest ssDNA or dsDNA [11], [12].

dsRNA is a unique product of virus infection and a powerful signal for host immune sensors that mount innate immune signaling pathways when its presence is detected [13]. The unique digestion of dsRNA by Lassa virus NP probably removes this pathogen-associated molecular pattern, and allows the virus to replicate without strong interference by the innate immune system. Structural features that confer this unique enzymatic specificity to NP were not apparent from previous crystal structures of the NP exonuclease, in which no substrate was bound. Here we present the crystal structure of the exonuclease domain of Lassa NP in complex with eight bases of dsRNA. This structure demonstrates that the unique substrate specificity is conferred by an extremely limited number of residues outside the enzymatic active site.

## Materials and Methods

### Protein production

The Lassa (Josiah strain) NP-exonuclease domain (residues 341–569, termed NPΔ340) was produced as previously described for the unbound NPΔ340 crystal structure[3]. Point mutations were introduced via site-directed mutagenesis with Phusion Hot Start polymerase (NEB).

### Crystallization

Deprotected, desalted, 8-base RNA oligonucleotides were purchased from Integrated DNA Technologies and delivered as lyophilized powder. One strand of the RNA was designed to include all purine residues (5′ GGAGGGAG 3′) and the other to include all pyrimidine residues (5′ CUCCCUCC 3′). Oligos were resuspended in nuclease-free water to a final concentration of 1 mM. Equal parts of sense and anti-sense oligos were combined and annealed at 65°C for 10 minutes before slow cooling to room temperature to yield dsRNA. Tris pH 8.0 and NaCl were added to the dsRNA to achieve final concentrations of 10 mM and 300 mM, respectively.

Crystals of the NPΔ340/E391A–8bp dsRNA complex were grown in 0.1 M Bis-Tris pH 6.5, 0.2 M MgCl_2_ hexahydrate and 25% PEG 3350. Crystals were cryoprotected with well solution containing 15% glycerol and flash-cooled in liquid nitrogen. Data were collected at the Advanced Photon Source, Beamline 19-ID.

### Data Processing and Structure Determination

**Figure 1:**
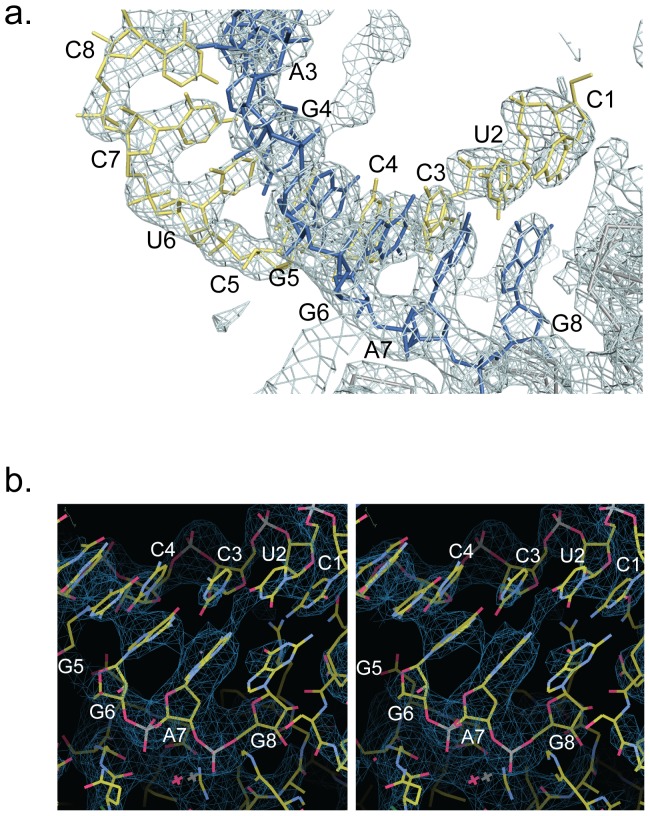
Electron density for the dsRNA. (a) Final 2Fo-Fc, contoured at +2.0σ, of the dsRNA bound to Lassa NP. The substrate strand, which leads into the active site is colored blue and the non-substrate strand is colored yellow. (b) Stereo view of a simulated-annealing composite omit map of final RNA density, contoured at +2.0σ.

Data were indexed, integrated, and scaled in space group P2_1_2_1_2 using HKL 2000 [14]. Structure determination of LASV NPΔ340/E391A–dsRNA by molecular replacement was carried out in PHENIX[15] using the uncomplexed NPΔ340 structure as a search model. One copy of NPΔ340 was found in the asymmetric unit. Initial Fo-Fc difference maps and 2Fo-Fc maps generated from the molecular replacement solution showed clear density for NPΔ340, as well as additional density for the dsRNA, which was not present in the search model (Figure 1). The dsRNA was built manually. Iterative cycles of model building were performed using Coot [16] and subsequent rounds of refinement were carried out using PHENIX. 5% of the reflections were set aside for *R*_free_ calculations. Simulated annealing was employed early in refinement to reduce model bias of the molecular replacement search model and simulating-annealing omit maps were used to confirm the RNA density. Custom hydrogen-bonding restraints between base-pairing nucleotides were generated using the ‘PDB to 3D restraints’ server [17] to maintain proper RNA base hydrogen-bonding distances. Initial models were uploaded to the server to identify hydrogen-bonding base pairs and to generate weighted ideal distance restraints of 2.9 Å between hydrogen-bond donors and acceptors of opposing nucleotide bases, which were then added to the refinement. The final NPΔ340/E391A–dsRNA structure has an *R*_free_ of 19.70% and R_work_ of 25.44% using all data to 2.9 Å (Table 1).

**Table 1:** Data collection, phasing and refinement statistics.

	NPΔ340/E391A–dsRNA complex
Data collection	
Space group	P2_1_2_1_2
Cell dimensions	
*a*, *b*, *c* (Å)	44.77, 84.31, 79.81
α, β, γ (°)	90, 90, 90
Wavelength (Å)	1.00
Resolution (Å)	39.9–2.91 (2.95–2.91)
*R*_merge_ (%)	7.4 (31.3)
Mean *I*/σ*(I)*	33.8 (2.4)
Completeness (%)	99.74 (99.65)
Redundancy	6.7 (6.6)
**Refinement**	
Resolution (Å)	39.9–2.91
No. unique reflections	7030
*R*_work_/*R*_free_	0.1970/0.2544
No. atoms	
Protein	1560
Ligand/ion	340
Water	5
*B* values	
Protein	71.77
Ligand/ion	86.90
Water	70.83
R.m.s deviations	
Bond lengths (Å)	0.014
Bond angles (°)	1.33

* Values in parentheses are for highest-resolution shell. _ENREF_3_ENREF_16_ENREF_17_ENREF_18_ENREF_19_ENREF_11_ENREF_13_ENREF_14_ENREF_9_ENREF_15_ENREF_13_ENREF_3

### Exonuclease Activity Assays

18 base DNA and RNA were purchased from IDT. Sequences of the oligonucleotides are as follows: RNA (sense) 5′ CAGGAUGUUGAUGCUGCU 3′; RNA (anti-sense) 5′ AGCAGCAUCAACAUCCUG 3′; DNA (anti-sense) 5′ AGCAGCATCAACATCCTG 3′. Oligonucleotides were labeled with P32 using protein nucleotide kinase according to the manufacturers directions (NEB). Standard reactions contained 50 nM wild-type or mutant NPΔ340 and 2 nM (labeled) and 10 nM (unlabeled) oligo(ribo)nucleotide. Sense and anti-sense RNA strands or, for hybrid experiments, sense RNA and anti-sense DNA were annealed at 65°C for 2 minutes and then cooled for 10 minutes at room temperature. Reactions were performed in 20 mM Tris pH 7.5, 150 mM NaCl, and 5 mM MgCl_2_. After incubation for 15 minutes, reactions were stopped with the addition of an equal volume of formamide loading buffer and boiled. The products were then analyzed in 18% polyacrylamide gels containing 8 M urea and buffered with 0.5x Tris-borate-EDTA. Gels were exposed overnight to a phosphorscreen and then imaged using a Typhoon phosphorimager.

## Results

### Structure of the exonuclease domain of Lassa NP bound to dsRNA

DEDDh exonucleases all have five invariant residues in the active site (Asp, Glu, Asp, Asp, and His) that are crucial for catalytic activity. In Lassa NP, these active site residues correspond to D389, E391, D466, D533 and H528. Wild-type Lassa NP rapidly digests dsRNA, and hence, it is not possible to stably crystallize wild-type NP with dsRNA for structural analysis. To increase the chances of obtaining a co-crystal structure of the exonuclease domain of Lassa NPΔ340 in complex with dsRNA, E391 was mutated to alanine. This mutation prevents the coordination of one of the two divalent cations required for the catalytic activity. As a result, NPΔ340E391A is able to bind dsRNA, but cannot catalyze the exonuclease reaction to digest it.

**Figure 2:**
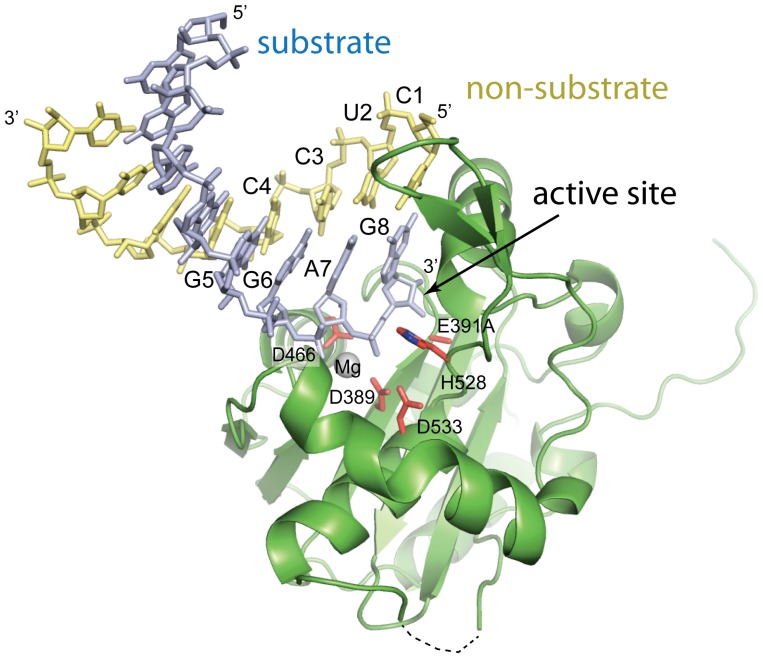
Structure of the exonuclease domain of Lassa NP in complex with 8 bases of dsRNA. A ribbon model of the immunosuppressive domain of Lassa NP is illustrated in green, with active site D, E, D, D, and H side chains drawn in red. We have arbitrarily modeled the digested strand as the purines and the non-digested strand as the pyrimidines. The substrate (purine) strand is colored purple and its 3′ terminal residue, G8, binds into the active site. The complementary, non-substrate (pyrimidine) strand is colored yellow, and its 5′ terminal residue is C1. One of the two divalent cations required for the exonuclease activity bound in the active site is modeled here as Mg. The second active site divalent cation is not bound due to required mutation of E391 to alanine to prevent digestion of the crystallized dsRNA.

Following molecular replacement using the unbound NPΔ340 structure (PDB code 3Q7B) as a search model, an Fo-Fc difference map showed positive electron density for a double-stranded substrate leading into the active site. By design, each individual strand of RNA is composed of either all-purine or all-pyrimidine bases. The single all-purine and all-pyrimidine strands were annealed to yield dsRNAs that each contain one all-purine and one all-pyrimidine strand. Digestion of dsRNA by Lassa NP is sequence-independent, and thus, there is equal likelihood for either the purine-containing or the pyrimidine-containing strand to be digested. Correspondingly, the electron density does not distinguish between the two. Hence, we have arbitrarily modeled the strand leading into the active site (the substrate strand) as the purine-containing strand and the complementary, non-substrate strand as the pyrimidine-containing strand (Figure 1 and Figure 2).

### Structure of the active site

**Figure 3:**
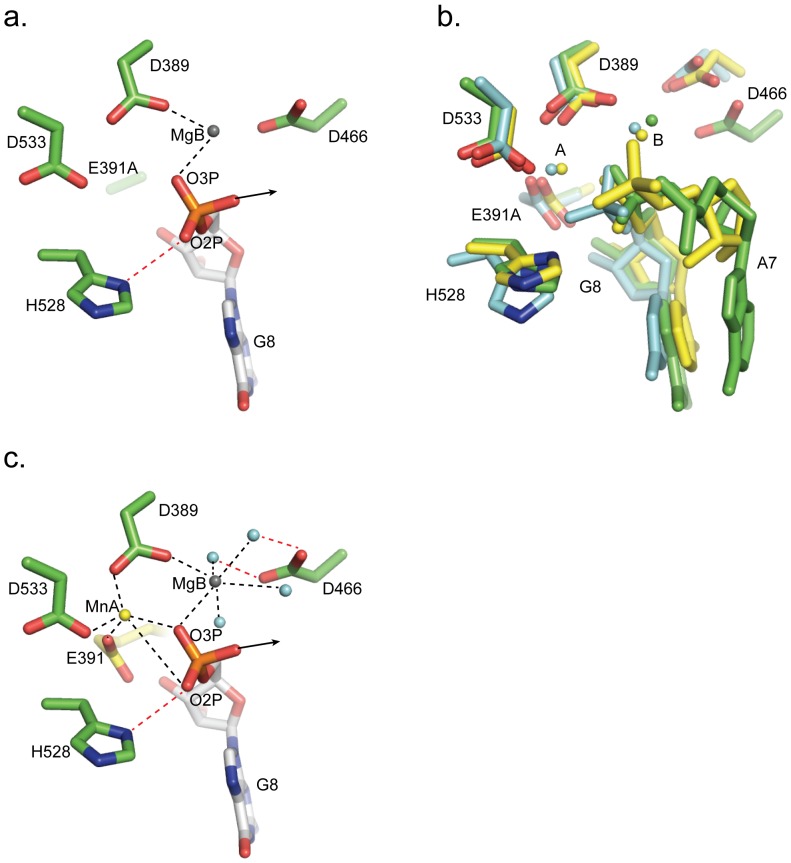
Structure of the active site. Carbons of Lassa NP residues are colored green. (a) The phosphate backbone of the terminal 3′ nucleotide (G8) is coordinated by a divalent cation (modeled here as a Mg ion, MgB) and by H528. MgB is further coordinated by D389. Due to mutation of E391 to alanine, the second catalytic divalent ion is not bound in the active site. (b) Comparison of the active site of Lassa NP E391A to the active sites of the DEDDh exonucleases ISG-20 (cyan, PDB code 1WLJ [5]) and ε186 (yellow, PDB code 1J54 [6]), which were determined in complex with mononucleotides. Both cations are apparent in the structures of ISG-20 and ε186. Amino acid numbering of the shared DEDDh motif is for Lassa NP. (c) A model of wild-type Lassa NP in complex with the terminal G8 mononucleotide. The crystallized ion MgB is drawn in grey. The position of E391 and the cation in site A are based on their positions in the unbound structure (PDB code 3Q7B [3]). Hydrogen bonds are colored red; coordination bonds are colored black; an arrow indicates the position of the next nucleotide in the digested strand. Expected water molecules, based on structural similarity to other DEDDh exonucleases, are drawn in pale blue.

As a result of the required mutation of E391 to alanine to prevent dsRNA digestion, only one of the two cations is present in the active site in the crystal structure (site B). This site B cation is modeled as a magnesium ion, because of the presence of 5 mM MgCl_2_ in the crystallization buffer, and is coordinated by D389 and by one of the phosphate oxygens of the 3′ terminal nucleotide of the digested RNA strand (G8) (Figure 3a). Alignment of the Lassa NP-dsRNA complex, with structures of ISG-20 [5] and ε186 [6], each in complex with a mononucleotide, shows that the positional arrangement of the active site nucleotide is extremely similar amongst all three DEDDh exonucleases (Figure 3b). In ISG-20 and ε186, the site B metal ions are coordinated by four water molecules. The site B ion Lassa NP may also be coordinated by water in a similar fashion, although water molecules are not visible at this resolution (Figure 3c). The site A metal ion missing in this mutant structure would likely be coordinated by the carboxylate oxygens of D389, E391 and D566, as was observed in our wild-type structure, and may also be coordinated by the phosphate oxygens of RNA base G8, as these exist in close proximity to the wild-type position of the site A metal.

**Figure 4:**
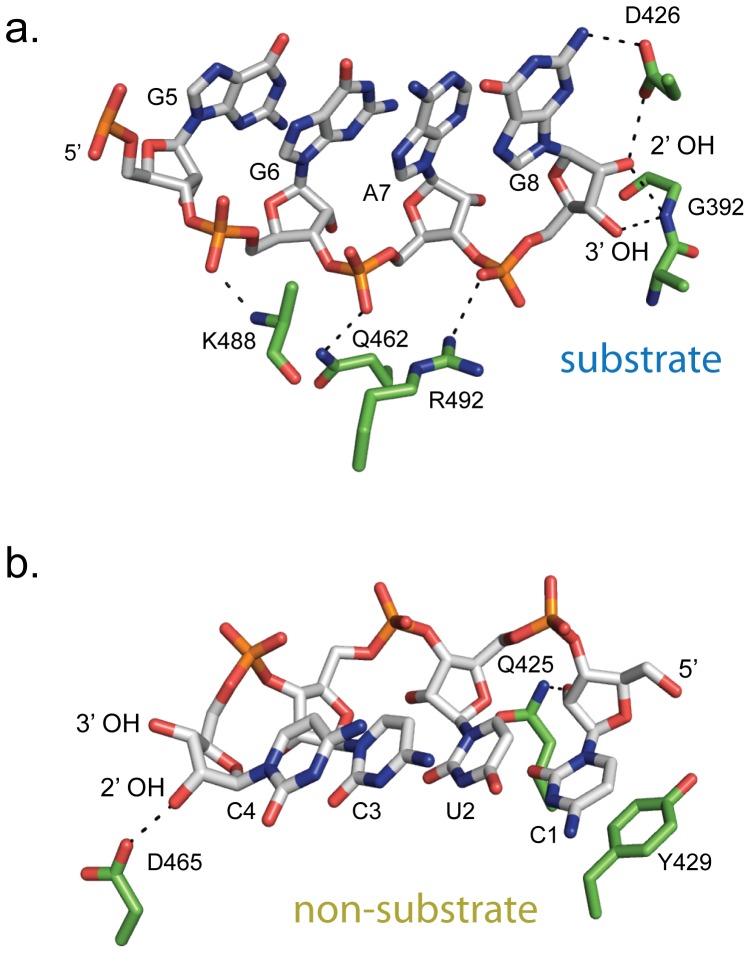
Protein-RNA contacts outside of the active site. (a) The substrate strand, which leads into the active site, forms hydrogen bonds to the NP through the phosphate backbone and by contacts to G8. D426 hydrogen bonds to its nitrogenous base, and the main chain nitrogen of G392 makes hydrogen bonds to the 2′ and 3′OH of its ribose sugar. (b) The non-substrate strand forms hydrogen bonds between the 2′OH of C1 and Q425, and the 2′OH of C4 and D465. There is also a base stacking interaction between C1 and Y429.

RNA base G8 is also observed to make several contacts to protein residues not included in the canonical DEDDh motif. In particular, the 2′OH of the ribose group and N2 of the nitrogenous base of G8 hydrogen bond to D426 of Lassa NP. Further, the 2′ and 3′ OH groups of G8 form hydrogen bonds to the main-chain nitrogen of G392 (Figure 4a).

### Protein:RNA contacts outside of the active site

The main-chain nitrogen of K488 and the side chain nitrogens of Q462 and R492 contact the phosphate oxygens of G6, A7 and G8 of the substrate strand, respectively (in the sequence ^5^′GGAGGG_6_A_7_G_8_ ^3^′) (Figure 4a).

The ribose 2′ oxygens of C1 and C4 of the complementary, non-substrate strand (in the sequence ^5^′C_1_UCC_4_CUCC^3^′) are bound by the side chains of Q425 and D465, respectively. In addition, the nucleotide base of C1 forms a π-π interaction with Y429 (Figure 4b). All contacts made by NP to either strand are consistent with the sequence-independent digestion of RNA by Lassa NP as they are made to the sugar phosphate backbone or to a ring structure that would be similar among all bases.

### Protein:RNA contacts required for exonuclease function

**Figure 5:**
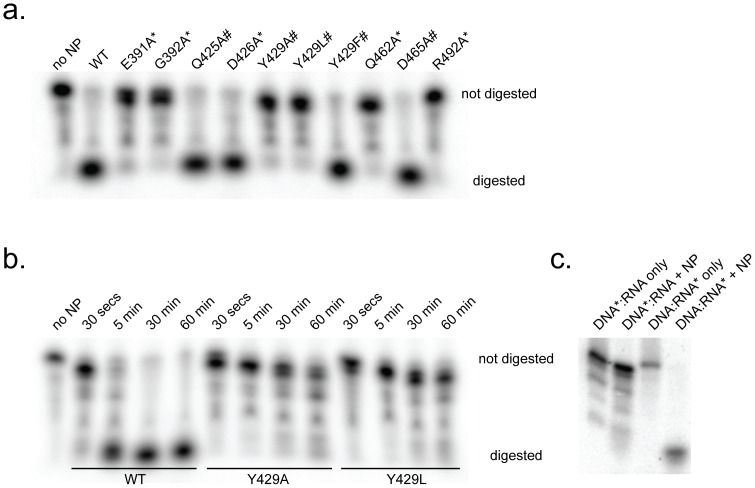
Effect of mutations on exonuclease activity. (a) Wild-type, full-length NP or the immunosuppressive domain of NP (NPΔ340) containing one of several point mutants were incubated with 18 bp blunt-ended dsRNA for 15 min, and products were analyzed by PAGE. NP residues that contact the digested strand are indicated with *; NP residues that contact the non-digested strand are indicated with #.’ (b) Time course of dsRNA digestion by wild-type (WT), and Y429A and Y429L mutant NPs. (c) Digestion of 18 bp hybrid DNA:RNA double-stranded oligonucleotides. The labeled strand is denoted by *.

In order to assess the relative contribution of each amino acid contact to RNA, we made alanine point mutations and tested each mutant for exonuclease function. As previously determined, mutation of E391, G392 and R492 to alanine prevents digestion of dsRNA [3] (Figure 5a). Here we show that mutation of Q462 to alanine and Y429 to alanine or leucine also prevents exonuclease activity. However, mutation of Y429 to phenylalanine (which retains the aromatic ring) and mutation of Q425, D426 and D465 to alanine have no effect on exonuclease activity.

The retention of exonuclease activity when Y429 is mutated to phenyalanine suggests that the stacking of the aromatic ring with base C1 is important. To determine if this base stacking was critical for RNA binding, or if it was necessary for efficient procession of the exonuclease activity, we performed a time-course study with WT, Y429A and Y429L Lassa NPs. When dsRNA is incubated with WT NP, the labeled RNA is digested within five minutes. By contrast, Y429A and Y429L-containing NPs show incomplete digestion even after 60 minutes (Figure 5b). It appears, however, that both of these mutations allow at least one nucleotide to be removed, suggesting that dsRNA binding itself is not disrupted but that the reaction cannot effectively proceed.

Our structure of NP bound to dsRNA demonstrates that contacts to the non-substrate strand are to the 2′OH of the ribose groups. However, based on our mutagenesis studies, these contacts appear to be non-essential. This observation led us to speculate that the non-substrate strand could be DNA as well as RNA. We thus generated a hybrid DNA:RNA double-stranded substrate in which either the DNA or RNA alone were 5′-labeled. When DNA was labeled and tracked, we saw no digestion. Conversely, when the RNA was labeled and tracked, there was digestion (Figure 5c). These results suggest that NP is capable of digesting a DNA:RNA hybrid, but only with DNA in the non-substrate strand position.

## Discussion

**Figure 6:**
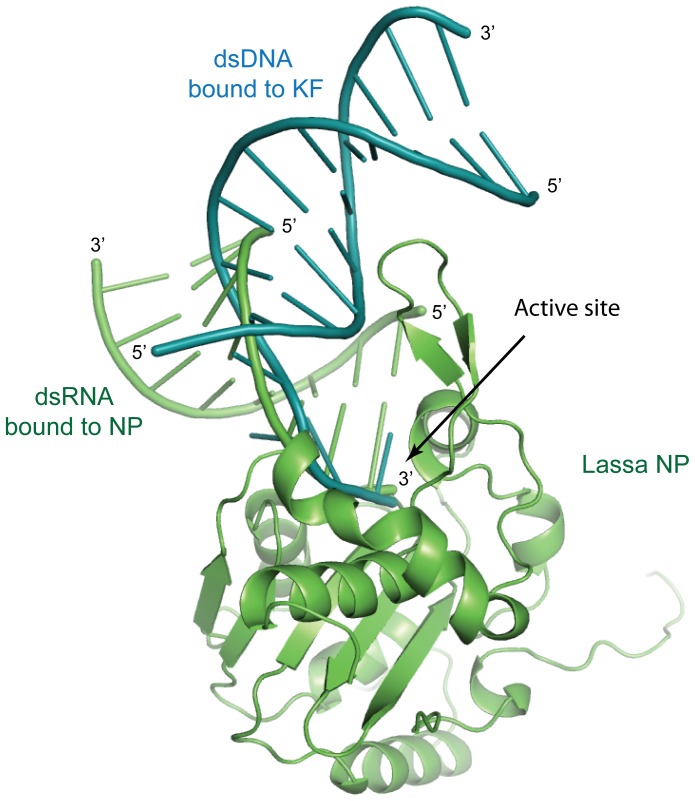
Comparison of double-stranded nucleic acids bound to Lassa NP and the exonuclease domain of the DNA pol I Klenow frament. Lassa NP bound to dsRNA (green) was aligned with the Klenow fragment exonuclease domain (KF) bound to dsDNA (teal, pdb code 1KLN [19]). The structures of Lassa NP and KF align with an r.m.s.d of 3.6 Å. For clarity, only the dsDNA of the KF complex is shown here. The dsRNA bound to Lassa NP remains based-paired throughout the entire length while the dsDNA bound to KF is split before entering the active site. Note that both 3′ ends align in the active site, but the 5′ end of the non-substrate dsRNA strand bound to Lassa NP remains base-paired, while the 5′ end of the non-substrate dsDNA strand bound to KF is split from the 3′ end and extends in the other direction.

There is little structural information available for exonuclease enzymes in complex with more than a single mononucleotide. Within the DEDDh exonuclease family, only Trex1 and 2 have been crystallized with an oligo: four bases of single-stranded DNA [7], [8], [18]. However, among the DEDDy family, which has a tyrosine residue rather than a histidine in the active site, the exonuclease domain of the Klenow fragment of DNA polymerase I (KF) has been crystallized with a longer, 12 bp double-stranded DNA [19]. Comparison of Lassa NP in complex with dsRNA and KF in complex with dsDNA demonstrates a different mode of binding between the two exonucleases. The dsRNA bound to Lassa NP remains base-paired throughout the entire length, including the final residue bound into the active site. By contrast, the two strands of the dsDNA bound to KF are split before entering the active site (Figure 6).

**Figure 7:**
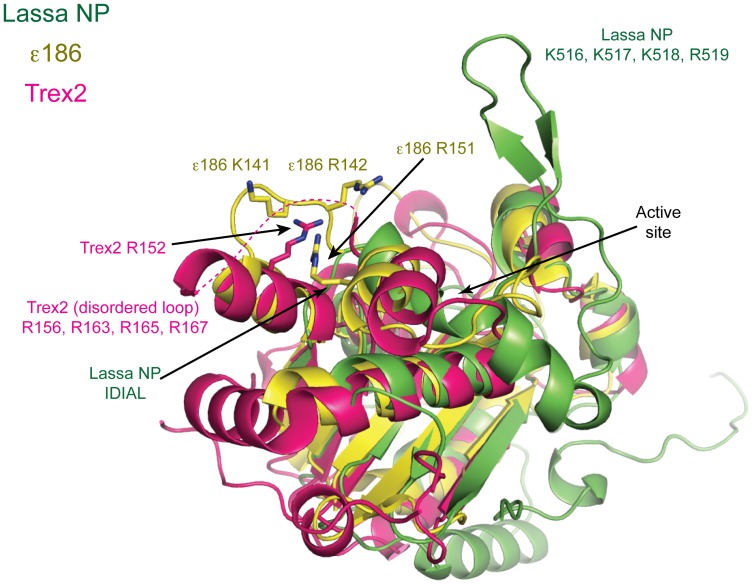
Location of the basic loop. Crystal structures of three DEDDh exonucleases are aligned. ε186 (PDB code 1J54 [6]) is illustrated in yellow, Trex2 (PDB code 1Y97 [9]) in magenta, and Lassa NP in green. All of these exonucleases contain a basic loop motif. In ε186, the basic motif (K141, R142, R151) lies above and to the right of the active site. In Trex 2, the basic loop is in the same general position, but all residues except for R152 are disordered and indicated by a dotted line. In Lassa NP, no such loop exists in this location and instead strand β5 and helix α5 are directly connected by an IDIAL hydrophobic sequence (residues I482 to L486). Instead, Lassa NP has a basic loop motif in a projecting “arm” located to the left of the active site rather than above and to the right. This loop contains K516, K517, K518 and R519, although the side chains are disordered in this structure.

KF, Trex 1 and 2 and ε186 are preferentially active towards unpaired 3′ termini [11], [12], [20]. These exonucleases contain a basic loop motif adjacent to the active site that may facilitate melting of duplex DNA in order to provide a suitable single-stranded 3′ end for the active site [6], [9], [19]. Certainly, mutation of this basic loop in KF and Trex2 reduces exonuclease activity [9], [21], suggesting a role for the motif in binding nucleic acid, particularly the non-substrate strand as it extends from the active site [9], [21]. We note that in Lassa NP, the site that is positionally equivalent to the basic loop of the other exonucleases is neither basic nor loop-shaped, but is instead much shorter and mostly hydrophobic (Figure 7). However, Lassa NP does encode a different basic loop located on the opposite side of the active site. Mutation of the basic residues in this loop also disrupts exonuclease activity [3]. This loop is in the correct position to interact with the non-substrate strand as it is “freed” from the duplex as the exonuclease reaction proceeds. Importantly, the “relocation” of the basic loop motif in Lassa NP may account for the differential mode of binding of duplex substrates seen between Lassa NP and the other exonucleases.

Mutational analysis of Lassa NP suggests that Y429 is the only critical contact to the non-substrate strand. Processive exonucleases such as Lassa NP require the stepwise procession of the substrate into and the subsequent removal of the product out of the active site. It is possible that interaction between Y429 and the RNA may facilitate this action. This is supported by the fact that mutation of Y429 to alanine or leucine permits binding, but greatly inhibits the digestion of RNA. Interestingly, other DEDDh exonucleases such as ISG-20 [5], Trex 1 [7], Trex 2 [18] and ε186 [6] do not contain a Tyr or other aromatic residue equivalent to Y429 of Lassa NP, and as previously mentioned, no other DEDDh exonuclease has been shown to have strict specificity for a double-stranded substrate. Thus, biochemical analysis and comparison to other exonuclease structures suggests that the specificity of Lassa NP for double-stranded substrates over single-stranded substrates is likely derived from the π-π stacking interaction between the tyrosine ring and the RNA base that previously complemented the one cleaved off in the active site.

**Figure 8:**
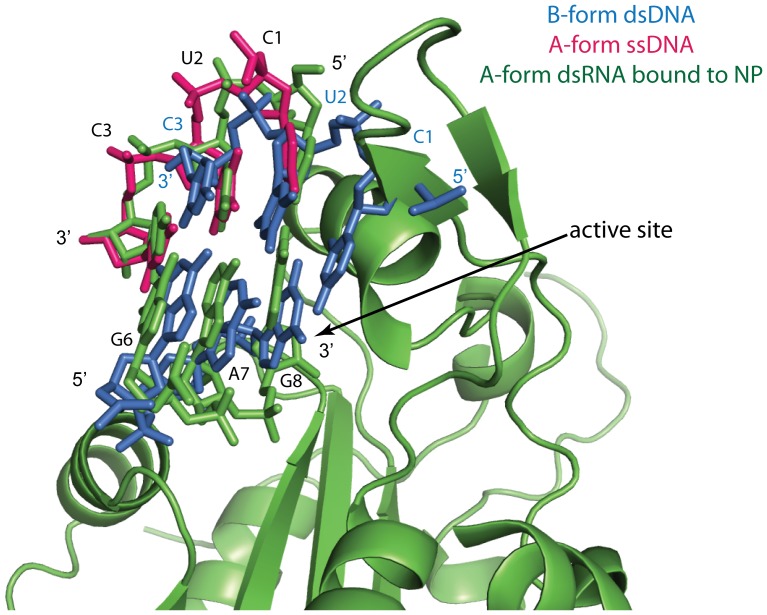
Modeling of different forms of DNA. Idealized B-form dsDNA (blue) and A-form ssDNA (magenta) were modeled and aligned with the dsRNA bound to Lassa NP (green). The 5′ terminal residue of the non-substrate strand (C1) in the B-form dsDNA clashes with Lassa NP, while A-form ssDNA does not.

Additionally, modeling of hybrid substrates suggests that Lassa NP can only efficiently bind to A-form duplex substrates. Modeling of B-form DNA demonstrates clashes would likely occur between the DNA and protein. However, there do not appear to be any clashes when A-form ssDNA is modeled as part of a hybrid RNA:DNA substrate (Figure 8). Notably, dsRNA is naturally found as A-form in the cell.

Further, a hydrogen bond between the RNA-specific 2′OH and the G392 in the active site could explain why the exonuclease activity of Lassa NP is specific for RNA over DNA in the substrate strand. This additional hydrogen bond, which would only be present in RNA, may help anchor the nucleotide within the active site. Although the contact is to the main-chain nitrogen and G392 is not part of the DEDDh motif, previous work demonstrated that mutation of G392 to alanine disrupts both the exonuclease function and the immunosuppressive function of NP [3]. The position of the main-chain nitrogen afforded by the uniquely flexible glycine seems to be important for proper orientation of the 3′ nucleotide of an incoming RNA strand relative to critical residues of the active site. Mutation of this residue to anything other than glycine would result in φ and ψ backbone dihedral angles outside allowable regions, unless several surrounding residues, including the active site D389 and E391, rearrange to accommodate it. Hence, mutation of G392 may force the active site into a non-functional arrangement. Curiously, the coronaviruses are the only other viruses known to encode a DEDDh exonuclease. SARS-CoV nsp14 was recently shown to be involved in RNA synthesis and genome fidelity [22], [23]. Notably, nsp14 will digest ssRNA and dsRNA, but not DNA, and also has a conserved glycine in the same position as Lassa NP G392. Although structural information is not yet available for nsp14, it is possible that in this enzyme as well, the φ and ψ backbone dihedral angles uniquely available to a glycine residue dictate the strict specificity for RNA over DNA.

In summary, we have identified two residues that lie outside the DEDDh active site motif that are essential for binding to and processing RNA. G392 appears to provide specificity for RNA within the active site, and is completely conserved amongst all arenaviruses and also the coronaviruses. Y429 appears to be important for the processive motion of the RNA, and is conserved among close relatives of Lassa such as LCMV and Lujo virus. Other arenaviruses, such as Junin and Machupo have a histidine at this position, which could provide a similar interaction with the RNA as a tyrosine.

The structure presented here suggests that the dsRNA-specific Lassa NP exonuclease enzyme binds to and processes double-stranded substrates using a mechanism distinct from other exonucleases. To our knowledge, Lassa NP is the only member of the DEDD families that has a basic loop to the left of the active site rather than to the right and is the only member thus far described that has strict specificity for double-stranded substrates. This work thus provides a template for design of anti-viral inhibitors of Lassa virus that could selectively target NP while leaving human DEDD exonucleases unaffected.
